# Supplementary material for: Uncovering the effects of interface-induced ordering of liquid on crystal growth using machine learning
Source: Nat Commun. 2020 Jun 26;11:3260. doi: 10.1038/s41467-020-16892-4 (PMC7319977; doi:10.1038/s41467-020-16892-4)
Supplement: Supplementary file 1 — Supplementary Information [file 41467_2020_16892_MOESM1_ESM.pdf]

Uncovering the effects of interface-induced ordering of liquid on crystal growth using Machine Learning

Freitas et al.

Supplementary Note 1: Machine Learning training

Note that all calculations described in this section were performed after relaxing the Molecular Dynamics (MD) snapshots (see Methods section for details).

Support Vector Machine

Given a set of data points $\mathbf{x}_i$ and corresponding labels y_i with two possible classes (± 1), the Support Vector Machine[1–3] (SVM) classifier finds the hyperplane of the form $\mathbf{w}.\mathbf{x} - b = 0$ that best separates these two classes, where $\mathbf{w}$ and b are the parameters that define this plane. Because data is often not perfectly separable by a hyperplane we have employed a soft-margin SVM, where data points on the wrong side of the hyperplane are penalized not but forbidden. In practice, this is done by performing the following minimization:

$$\min_{\mathbf{w},b,\xi} \left[\frac{1}{2}\|\mathbf{w}\|^2 + C\sum_{i=1}^{n}\xi_i \right] \tag{1}$$

subject to the constraint $y_i(\mathbf{w}\cdot\mathbf{x}_i - b) \geq 1 - \xi_i$ for $i = 1, 2, \ldots, n$ and $\xi_i \geq 0$. The parameters ξ_i control how far from the hyperplane are the misclassified examples, and C is the penalty term of the classification error.

Hyperparameter tuning

The set of radial structure functions[4,5] $\mathcal{G}_i(r)$ used as the local-structure fingerprint was selected based on a hyper-parameter tuning procedure using grid search[6]. The hyperparameters optimized were σ, the radius cutoff for neighbors considered in the calculation (r_{cut}), and the separation Δr between the r values employed. Using a SVM with no kernel (also known as linear SVM) and a penalty parameter of $C = 1.0$ we trained classifiers using random sets of $2,000$ examples and evaluated the accuracy of each classifier using a separate test set of $10,000$ data points. The data was extracted from the crystal growth simulations at 1500 K.

Supplementary Figure[*] 1a shows the grid-search results for the maximum value of r considered ($r_{\max}$) and different values of Δr. In practice we have chosen $r_{\text{cut}} = r_{\max} + 2\sigma$. Shown in Supplementary Figure 1b is the grid-search results for σ, the accuracy of the highest-complexity model considered is also shown for comparison. The chosen values of the hyperparameters are: $\sigma = 0.5$ Å, $\Delta r = 0.40$ Å, and $r_{\text{cut}} = 10.8$ Å (or, equivalently, $r_{\max} = 10.0$ Å), colored in black in Supplementary Figure 1.

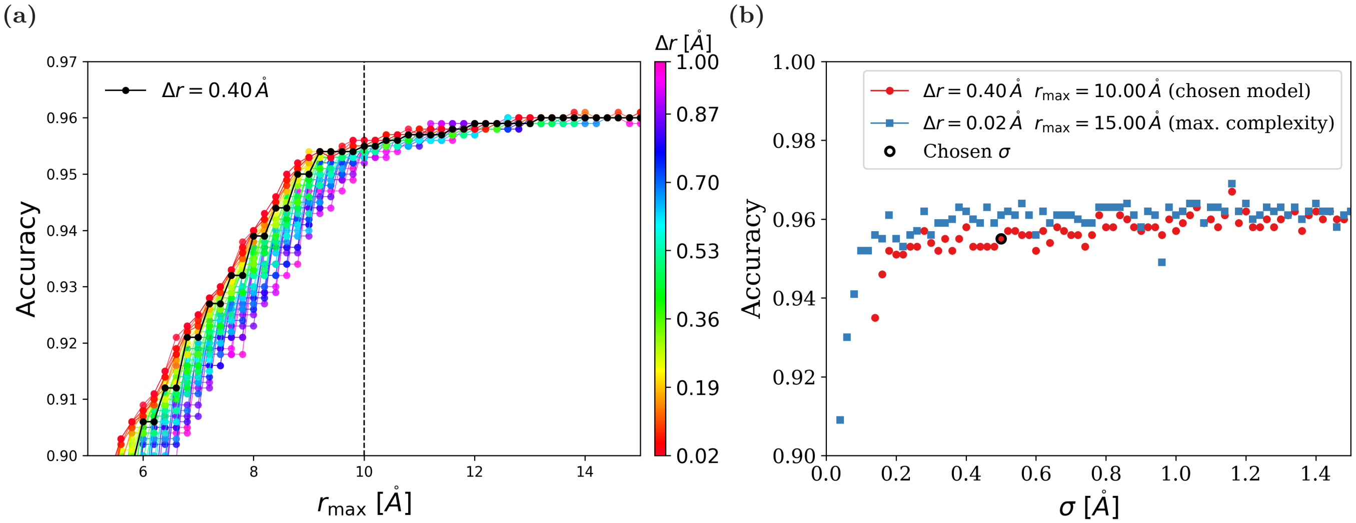

Supplementary Figure 1: Grid-search hyperparameter tuning for model feature selection. **(a)** Accuracy of models with varying $r_{\max}$ and Δr for $\sigma = 0.5$ Å. The chosen model has $\Delta r = 0.40$ Å (black circles) and $r_{\max} = 10$ Å (vertical dashed line). **(b)** Accuracy of models with different values of σ. Blue squares show the accuracy of the highest-complexity model considered (i.e. largest number of features) for comparison. Red circles show the accuracy of the chosen values of Δr and $r_{\max}$ for different values of σ.

The improvement of classification accuracy with the use of kernels was negligible and agreed within the error bars with the accuracy of linear SVM (i.e. no kernel SVM). The statistical origins of this result (as explained in refs. 8 and 9) is that the dimensionality of the data (number of features) was enough that the separating hyperplane with kernel SVM was

[*]Unless otherwise noted, all figures were created using `matplotlib`[7].

a very smooth surface. The physical origins of this result (as explained in refs. 5 and 10) is that once enough information about the local structure is provided in the form of a number of features the discrimination between different structures becomes easy to perform with linear algorithms. It is shown in ref. 10 that even the use of artificial neural networks does not improve the accuracy of the classification task. In fact, ref. 10 shows that even very rudimentary features can be employed as long as enough of them are provided in order to describe the local structure adequately.

Learning and validation curves

The classification error penalty parameter C (Supplementary Equation (1)) was found by using a grid-search algorithm. For each value of $C \in [10^{-3}, 10^{+5}]$ a five-fold cross-validation procedure was performed in a random set of $12,500$ data points. The validation curve for C is shown in Supplementary Figure 2a, where we see that the accuracy of the models are independent of C for a wide range of values. Thus, for all results presented in the paper we pick $C = 1.0$, shown as the dashed line in Supplementary Figure 2a.

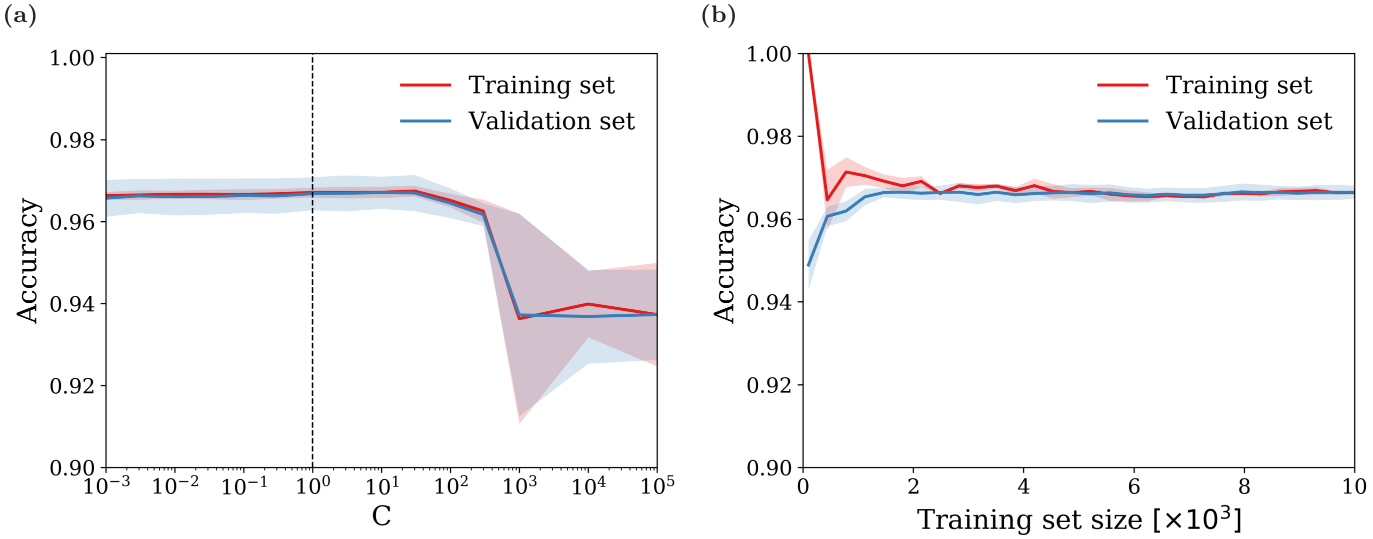

Supplementary Figure 2: **(a)** Validation curve for SVM parameter C. Dashed line ($C = 1.0$) marks the value employed in calculations discussed in the paper. Error bars were computed using a five-fold cross-validation procedure. **(b)** Learning curve with error bars computed using a five-fold cross-validation procedure.

The size of the training set was determined by computing the learning curve (with $C = 1.0$), as shown in Supplementary Figure 2b. The error bars were computed using five-fold cross validation. From Supplementary Figure 2b we see that a training set consisting of a few thousand data points is enough to bring the accuracy of the model close to the plateau in this curve. Because of the abundance of data points available we pick a training set size of $10,000$ to train the classifiers.

Supplementary Note 2: Labeling

The labeling of the data consists in determining whether atoms are in the bulk liquid, bulk crystal, or currently undergoing crystallization (i.e. close to the activated state at the top of the energy barrier in Fig. 2a in the paper). In order to determine the atoms' label we have used the time-evolution of the $\alpha_i(t)$ parameter[11], as described below. Notice that all calculations described in this section were performed after relaxing the MD snapshots (see Methods section for more details).

The $\alpha_i(t)$ parameter

Consider the $N_\mathrm{b}(i)$ first neighbors of the ith atom in the system. In practice we have considered as first neighbors all atoms within 3 Å of the ith atom. The distance of 3 Å was selected based on the radial distribution function of the bulk liquid and bulk solid phases: 3 Å is the first minimum of the radial distribution function of the bulk liquid phase, as shown in Supplementary Figure 3.

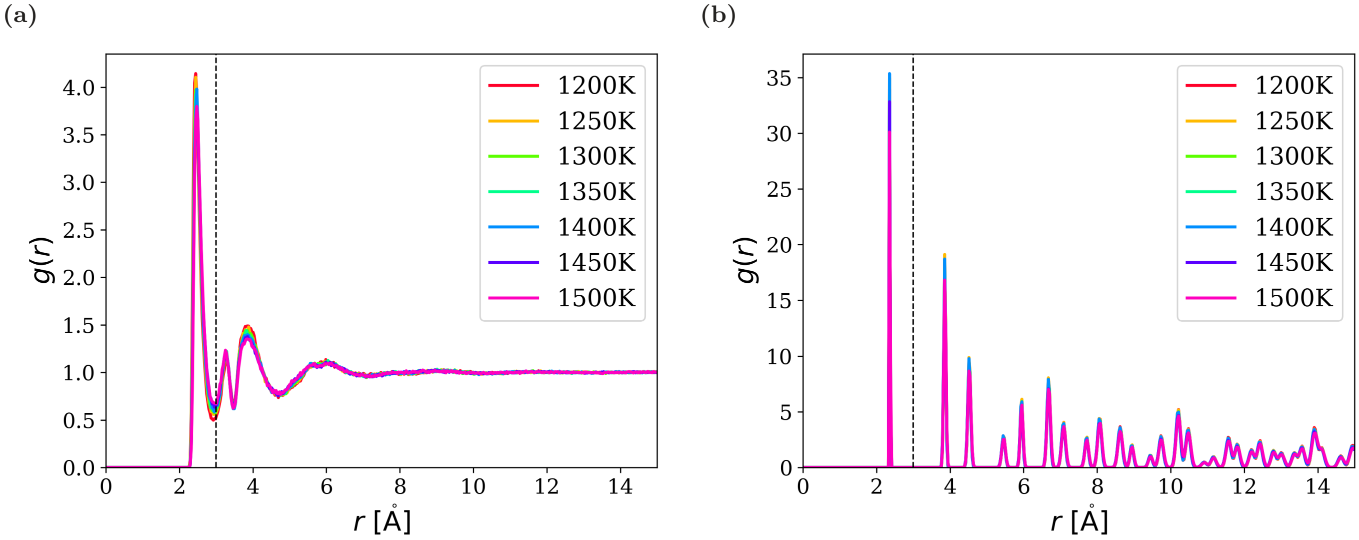

Supplementary Figure 3: **(a)** Radial distribution function for liquid silicon. The dashed line marks the cutoff radius of 3.0 Å (i.e. the first minimum of the curve) for the calculation of $\alpha_i(t)$. **(b)** Radial distribution function for crystalline silicon. The sharp peaks are a result of the system relaxation performed before computing structural properties.

For every atom i we have computed the scalars:

$$q_{\ell m}(i) = \frac{1}{N_\mathrm{b}(i)} \sum_{j=1}^{N_\mathrm{b}(i)} Y_\ell^m(\theta_{ij}, \phi_{ij}),$$

where $Y_\ell^m(\theta_{ij}, \phi_{ij})$ are spherical harmonics, θ_{ij} is the polar angle between the pair of atoms i and j, and ϕ_{ij} is the azimuthal angle between the pair of atoms i and j. With these values we constructed the following vector for each atom:

$$\bar{\mathbf{q}}_\ell(i) = \Big[q_{\ell-\ell}(i), q_{\ell-(\ell-1)}(i), \ldots, q_{\ell(\ell-1)}(i), q_{\ell\ell}(i)\Big],$$

and finally the vector $\mathbf{q}_\ell(i)$ was built by normalizing $\bar{\mathbf{q}}_\ell(i)$:

$$\mathbf{q}_\ell(i) = \frac{\bar{\mathbf{q}}_\ell(i)}{\sqrt{\bar{\mathbf{q}}_\ell^*(i) \cdot \bar{\mathbf{q}}_\ell(i)}}.$$

Vector $\mathbf{q}_\ell(i)$ can be seen as a descriptor of the structure of the first neighbors of atom i. Thus, if $\mathbf{q}_\ell(i)$ and $\mathbf{q}_\ell(j)$ are aligned with each other the structure surrounding atoms i and j are similar, as we would expect in a crystal. Hence, if j is a first neighbor of i then for $\mathbf{q}_\ell(i) \cdot \mathbf{q}_\ell^*(j) \geq q_\mathrm{cut}$ we say atoms i and j have a "crystal-like" bond.

In order to determine the spherical harmonics degree ℓ to be used in the calculation of $\mathbf{q}_\ell(i)$ we have analyzed the density distribution of $\mathbf{q}_\ell(i) \cdot \mathbf{q}_\ell^*(j)$ for liquid and solid phases for $\ell = 1, 2, \ldots, 10$. From the density distribution it became clear that $\ell = 6$ and $\ell = 7$ have liquid and solid density distributions that overlap the least, with $\ell = 6$ being slightly

better. Thus, we have chosen to employ $\ell = 6$ in our calculations with a cutoff value of $q_{\rm cut} = 0.80$. This choice of parameters identified crystal atoms with 100% accuracy, while liquid atoms were identified with accuracy better than 99.5% for all temperatures from 1200 K to 1500 K.

Finally, for an atom i in a MD snapshot at time t the parameter[11] $\alpha_i(t)$ is the fraction of its $N_{\rm b}(i)$ first neighbors with $\mathbf{q}_\ell(i) \cdot \mathbf{q}^*_\ell(j) \geq q_{\rm cut}$, i.e. $\alpha_i(t)$ is the fraction of "crystal-like" bonds made by atom i at time t.

Encoding the dynamics

The time evolution of $\alpha_i(t)$ is used to label the state of the atom (liquid, crystal, or crystallizing). Figure 4a illustrates the labeling process described in the Methods section "Encoding atomic dynamics (ML labeling)". In Supplementary Figure 4b we show $\alpha_i(t)$ and $\bar{\alpha}_i(t)$ for an atom undergoing crystallization at $t \approx 1.5$ ns during a simulation at $T = 1500$ K. Figure 4c focus on the time interval around the crystallization event of the same atom. In these figures the curve for $\alpha_i(t)$ is shown in gray, the window average $\bar{\alpha}_i(t)$ is shown in black, and the background is color coded to reflect the states illustrated in Supplementary Figure 4a.

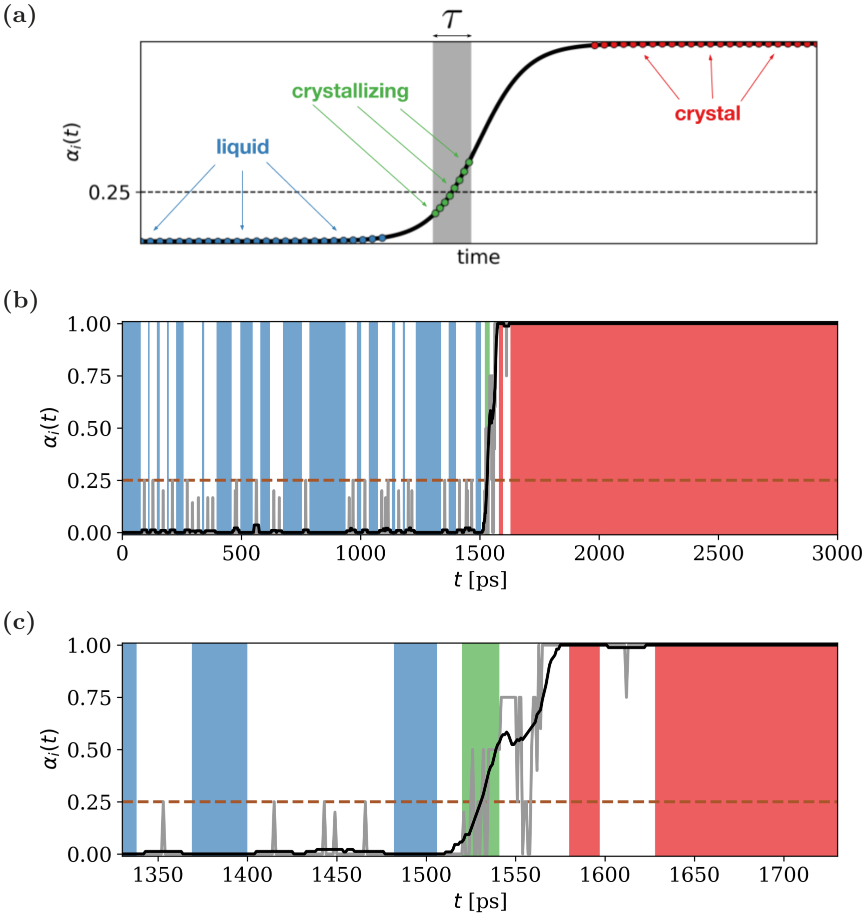

Supplementary Figure 4: **(a)** Illustration of the labeling process for the atomic dynamics as described by $\alpha_i(t)$. We have taken $\tau = 20$ ps. **(b)** Representative plot of $\alpha_i(t)$ for an atom undergoing crystallization at $t \approx 1.5$ ns. The gray line is $\alpha_i(t)$ while the black line is the window average $\bar{\alpha}_i(t)$. Background color represent the label of the atomic state at that time, blue for $y = -1$ (liquid), green for $y = 1$ (crystallizing), and red for atoms in the crystal state (used only in the PCA and tSNE analysis). The horizontal dashed line at $\alpha_i = 0.25$ is used as a threshold for labeling, as described in the text. **(c)** Same as previous plot, but with time interval focused around the crystallization event.

Figure 4c indicates that $\bar{\alpha}_i(t)$ does not fluctuate much when the atom is in the liquid state, making it easy to detect liquid atoms as those with $\bar{\alpha}_i(t) \approx 0$. Defining crystallizing atoms is more complicated due to the large fluctuations in $\alpha_i(t)$ during the liquid-to-solid transition. Atoms undergoing crystallization are defined as those atoms within a 20 ps window around the time where $\bar{\alpha}_i(t) = 0.25$. The window length and the $\bar{\alpha}_i(t)$ threshold are chosen in conjunction, with the goal of capturing the moment an atom leaves the liquid state. Ideally, we would like to capture a single time snapshot at which the atom leaves the liquid state. But, because of the presence of thermal fluctuations this is not possible: the liquid state (or any other state of the system) is defined as a statistical distribution of configurations (or ensemble), not a single state. Thus, we choose the window length (20 ps) to be small compared to the time it takes for the atom to undergo

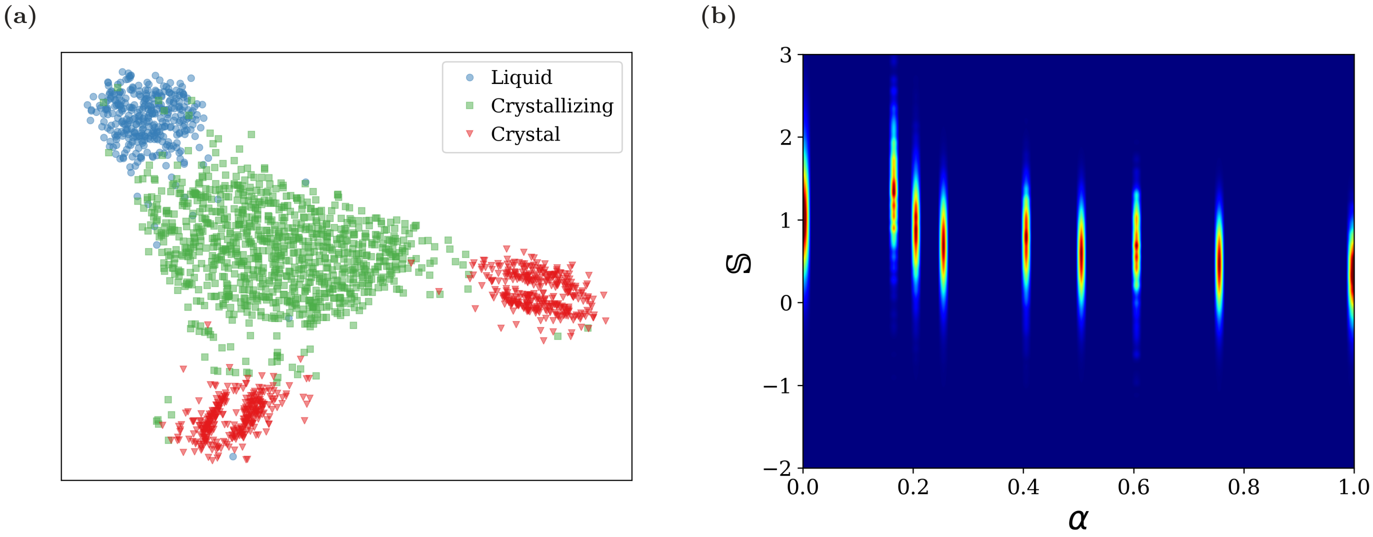

Supplementary Figure 5: **(a)** Two-component tSNE plot showing how the structural features capture the labeling of the crystallization dynamics. Notice that the labels (represented here as colors) were not use by tSNE, since tSNE is a unsupervised method. The different crystal regions correspond to atoms belonging to a perfect diamond structure and atoms belonging to stacking faults. **(b)** Histogram of softness as a function of α. Note that the value of α is discrete because it represents the fraction of crystal-like bonds.

complete crystallization. Similarly, the $\bar{\alpha}_i(t)$ threshold (0.25) is visually chosen as to capture states just after $\bar{\alpha}_i(t)$ starts increasing from its ≈ 0 value in the liquid state.

Due to the nature of the thermal fluctuations, it is possible for $\bar{\alpha}_i(t)$ to cross the 0.25 mark many times before reaching the crystal state. To avoid marking these spurious events as crystallizing events we employ an algorithm that first detects when $\bar{\alpha}_i(t_1) = 0.50$, and from t_1 it backtracks to the last occurrence of $\bar{\alpha}_i(t) = 0.25$ before t_1. Effectively, the algorithm first detects whether the thermal fluctuations were sufficient to lead the atom to the crystal state and, if successful, it finds the point where that atom last left the liquid state to join the crystal.

Connections between local-structure and dynamics

A set of 21 radial structure functions were used as local-structure fingerprint $\mathbf{x}_i = [\mathcal{G}_i(r_1), \mathcal{G}_i(r_2), \ldots, \mathcal{G}_i(r_{21})]$. Now that we have labeled the atomic dynamics by determining the atomic state (as described in the last section), it is possible to investigate if the distribution of the local-structure fingerprint $\mathbf{x}_i$ in this 21-dimensional space ($\mathbb{R}^{21}$) reflects the atomic dynamics. This can be done with dimensionality reduction techniques such as the t-Distributed Stochastic Neighbor Embedding[2,12,13] (tSNE) method. tSNE's goal is to generate a faithful representation of the $\mathbb{R}^{21}$-distribution of $\mathbf{x}_i$ in two dimensions. This non-linear unsupervised algorithm (i.e. an algorithm that only uses as input the $\mathbf{x}_i$ vectors) performs different transformations in different regions of $\mathbb{R}^{21}$ to find a balance between the local and global aspects of the $\mathbf{x}_i$ distribution.

In Supplementary Figure 5a we show the tSNE plot for 2,000 local-structure fingerprint vectors randomly chosen and equally distributed between the three possible labels (liquid, crystallizing, and crystal). The tSNE plot was generated with a perplexity value of 200. Before applying the tSNE algorithm we first passed the raw $\mathbf{x}_i$ vectors through a PCA (Principal Component Analysis[2,6]) filter. Only the first 12 PCA components were passed to tSNE because our analysis indicated that these components explained more than 99% of the variance in the data.

After the tSNE analysis was performed we identified the tSNE output with the dynamic labels computed through the analysis of $\alpha_i(t)$, shown in Supplementary Figure 5a with different colors. It is clear from this figure that the structural fingerprint $\mathbf{x}_i$ is capable of discerning between the three atomic state labels. It is also interesting to notice that the crystal state was further separated into two distinct groups. Such separation into two groups was also observed before, in the calculation of $\alpha_i(t)$ and also in the PCA analysis results shown in Fig. 3b of the paper. The difference between the two groups can be easily determined (through $\alpha_i(t)$, as described in Methods) to be due to the presence of stacking faults in the system, which change the local structure of atoms inside the defect to hexagonal diamond instead of cubic diamond. Upon close inspection we also notice that each crystal group (with and without stacking faults) seems to also be separated into two subgroups. The presence of these subgroups has not been observed in the analysis of the $\alpha_i(t)$ parameter or the PCA analysis (Fig. 3b of the paper), and thus the determination of its structural origin is difficult to perform. Our hypothesis is that these subgroups exist due to the presence of other structural defects observed in the crystallite, such as

dislocations (Supplementary Figure 8b) and vacancies.

Correlations between α and softness $\mathbb{S}$: liquid ordering

In the paper we demonstrated that solid-liquid interfaces affect the mobility the nearby liquid atoms during crystallization. The consequences of this change in mobility on the crystallization kinetics are shown to be well captured by softness ($\mathbb{S}$), and thus we concluded that the observed change in mobility is caused by an associated change in the liquid structure. In this section we establish that the liquid structure changes in such a way as to become more ordered.

Softness is computed using information about the local structure surrounding the crystallizing atom. It is difficult, however, to use $\mathbb{S}$ to show that the liquid structure becomes more ordered because $\mathbb{S}$ also accounts for the local structure due to the nearby crystal. This occurs because we use information of all neighbors of the crystallizing atom within $r_{\text{cut}} = 10.8\,\text{Å}$. However, the parameter α is a purely structural parameter designed to measure the degree of order around an atom, and it does so by using only information of its first neighbors (i.e. atoms within $3\,\text{Å}$).

In Supplementary Figure 5b we plot the density distribution of $\mathbb{S}$ for each value of α for all the crystallizing atoms. Supplementary Figure 5b shows that $\mathbb{S}$ and α are inversely correlated, since an increase in alpha (i.e. an increase in liquid ordering) leads to smaller values of $\mathbb{S}$. Notice that this is exactly the relationship illustrated in Fig. 8 and explained in the Discussion section: an increase in IIO is accompanied by a decrease in liquid mobility, which occurs as $\mathbb{S}$ decreases.

Supplementary Note 3: Machine-Learning model physical rationale

Model independence on the training set temperature.

For the local-structure dependent (LSD) model to be physically consistent, it is important that its features and parameters do not depend on the conditions under which the SVM classifier is trained. This was achieved by two deliberate choices we made. In Supplementary Note 4 we discuss the first choice: train on data obtained during steady-state growth conditions. This choice should remove the dependency on the specific time of the simulation at which data was collected, making the model valid for an arbitrary crystallite growing under steady-state conditions. In this section we show the implications of the second choice made: training the model on MD snapshots after a brief period of structural relaxation as described in the Methods section "Crystal growth simulations". By relaxing the system we are quenching it into its local inherent structure[14], providing a single value of $\mathbb{S}$. This approach curbs the effects of structural distortions due to thermal fluctuations and it allows us to sample the true (or inherent) local structure of the crystallizing atoms.

To test the independence of the model on the training set temperature we trained SVM classifiers at different temperatures, using data sets obtained from simulations at temperatures ranging from 1300 K to 1500 K in 50 K intervals. Each data set had $2,500$ randomly chosen structural fingerprints ($\mathbf{x}_i$) from each class ($y_i = \pm 1$). Then we tested the accuracy of each model on a test set consisting of $20,000$ randomly selected data points obtained from a crystal growth simulation at 1500 K. The accuracy of all SVM classifiers were higher than 96%, with a total variation of 0.4%.

The independence of the SVM classifier's accuracy is a good indicator of the physical consistency of the model. But, the most rigorous test is to verify if the parameters of the LSD model $r(T, \mathbb{S})$ depend on the training temperature, i.e. the activation energy $\Delta E_{\mathrm{a}}(\mathbb{S})$, the prefactor $k_0(\mathbb{S})$, and the interface free energy $\gamma(\mathbb{S})$. In Supplementary Figure 6 we show that these three parameters (which uniquely define the model's dependence on $\mathbb{S}$) can be measured with the SVM classifier trained at any temperature: the measured values agree with each other within the error bars. These figures can be compared directly to Figs. 5b, 5c, and 6a of the paper.

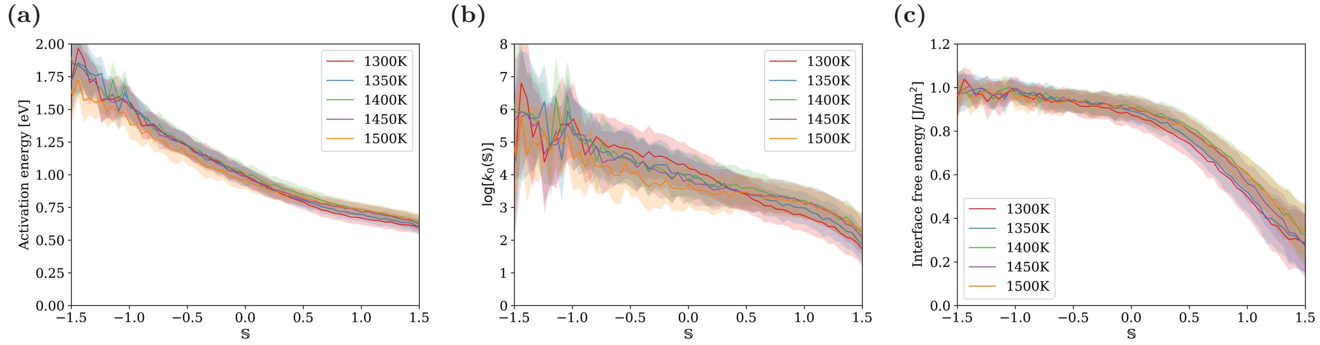

Supplementary Figure 6: Parameters of the local-structure dependent model $r(T, \mathbb{S})$ as computed training the SVM classifier with data obtained at different temperatures. **(a)** Activation energy $\Delta E_{\mathrm{a}}(\mathbb{S})$. **(b)** Arrhenius prefactor $k_0(\mathbb{S})$. **(c)** Interface free energy $\gamma(\mathbb{S})$. Together these parameters uniquely define the local-structure dependent model. Because they do not depend on the training set temperature, the $r(T, \mathbb{S})$ model is physically consistent.

Hence, we believe that the final LSD model does have physical reality. The source of this quality comes from including atomistic information about crystallization mechanisms in creating the $r(T, \mathbb{S})$ model, resulting in a type of model much different from the fitting of a phenomenological model to the observed data (as it has been the case with the Wilson-Frenkel model since its inception).

Physical interpretation of the learning process

In the SVM learning process each label y_i was characterized by a set of features $\mathbf{x}_i = \left[\mathcal{G}_i(r_1), \mathcal{G}_i(r_2), \ldots, \mathcal{G}_i(r_{21})\right]$ where

$$\mathcal{G}_i(r) = \sum_{j=1}^{n(i)} \exp\left[-(r_{ij} - r)^2 / 2\sigma^2 \right],$$

with $n(i)$ being the number of neighbors of atom i within a cutoff radius r_{cut}. The functions $\mathcal{G}_i(r)$ are known as radial structure functions and have a clear physical interpretation: $\mathcal{G}_i(r)$ counts the number of neighbors of atom i at a distance r in such way that if a neighbor j is at a distance $r_{ij} \neq r$ it will be counted as a fraction of a neighbor, with this fraction decreasing with $|r_{ij} - r|$ and the rate of decrease determined by σ. Thus, there exists an exact relationship[4,5,10] between

$\mathcal{G}_i(r)$ and the radial distribution function $g(r)$:

$$g(r) = \lim_{\sigma \to 0} \left[\frac{\mathcal{G}(r;\sigma)}{\rho\Omega(r,\sigma)} \right],$$

where ρ is the system density, $\Omega(r,\sigma)$ is the volume of a spherical shell of radius r and thickness 2σ, and $\mathcal{G}(r;\sigma) = \langle \mathcal{G}_i(r;\sigma) \rangle$, where the average is taken over all atoms.

In order to gain insight on the learning process of our model[5,10] we trained a SVM classifier using small σ and Δr, we chose $\sigma = 0.10$ Å and $\Delta r = 0.2$ Å. With this fine-grain resolution of the features we trained SVM classifiers using only one feature at the time, i.e. $\mathbf{x}_i = [\mathcal{G}_i(r_n)]$, and $C = 1.0$. In Supplementary Figure 7 we plot the accuracy of each of these models versus the value of $r = r_n$ used as feature, shown as the red line (with values marked on the left vertical axis). We observe that the accuracy of the features $\mathcal{G}(r_n)$ is marked by well-defined cycles ranging from the baseline value of 50% (i.e. not better than random guessing) to remarkable maximum values of up to 75% accuracy for a single feature.

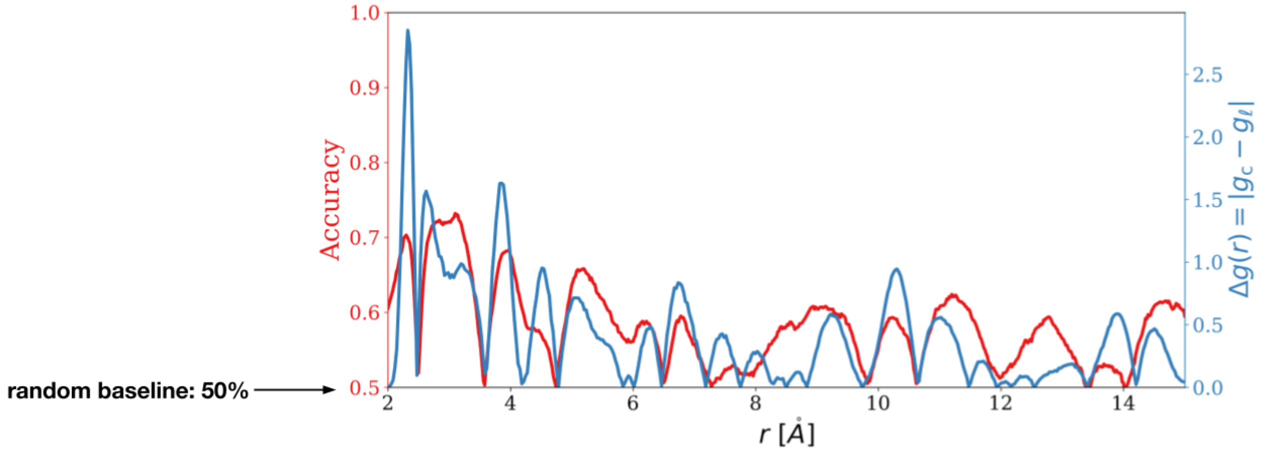

Supplementary Figure 7: Red line is the accuracy of SVM models trained with a single feature $\mathcal{G}(r)$ where $\sigma = 0.1$ Å, with values marked on the left axis. Blue line correspond to $\Delta g(r) = |g_c(r) - g_\ell(r)|$, i.e. the absolute value of the difference in radial distribution functions of the crystal and liquid phases. The accuracy of each single feature cycles in synchrony with $\Delta g(r)$ up to distances of 15 Å, suggesting that the ML model learns the distinction between liquid atoms and crystallizing atoms through a complicated assimilation process that takes into account how far the density of neighbors is from the liquid and crystal states.

The origin of this cyclic behavior can be understood by comparing the accuracy dependence on r to the radial distribution function $g(r)$. The radial distribution function of the crystal $g_c(r)$ or the liquid $g_\ell(r)$ do not match the cycles observed in Supplementary Figure 7. But, their absolute difference $\Delta g(r) = |g_c(r) - g_\ell(r)|$ seem to cycle in synchrony with the accuracy, as shown in Supplementary Figure 7 in the blue line (with values marked on the right vertical axis).

Together, the observations above suggest that our ML model learns the distinction between liquid and crystallizing atoms by measuring how far the density of neighbors is from their mean values in the liquid state. Consequently, if $\Delta g(r_n) = 0$ the accuracy of the model trained on feature $\mathbf{x}_i = [\mathcal{G}_i(r_n)]$ is the baseline (i.e. random guessing) since there is no way to distinguish between liquid and crystal at those values of r.

It is also interesting to notice in Supplementary Figure 7 that the accuracy of models trained at distances $r_n > 3$ Å is as high as 68%, and it is often above 60%. This observation is important because the labeling of the atomic states was performed using structural information up to 3 Å cutoff. Therefore, Supplementary Figure 7 shows that the ML classifier is not trivially learning our labeling process, but instead it is assimilating complicated information about the dynamics of atomic mechanisms taking place at distances as far as 15 Å.

Finally, an analysis of the linear correlation of the features was performed. We computed how much of the variance in the data is explained by each of the 21 components of the PCA spectrum (one for each dimension of x_i), then the components were ranked by their contribution to the variance. This analysis shows that 99% of the variance is recovered with the 12 first components, while 90% of variance requires at least seven components, and one component contained 42% of the variance. Given the analysis presented above on $\Delta g(r)$ these results are somewhat expected: simple features like average density will play a big role in classification of structures during a solid-liquid transition. Thus, while some linear correlation is present, it is far from being a dominant characteristic (such as to allow a much lower dimensional space to be used), and multidimensional feature vectors are indeed necessary to reproduce the accuracy observed.

Truncating softness at $\mathbb{S} = 1.5$

In this section we will address some details on how the growth rates as a function of $\mathbb{S}$ (shown in Fig. 4b of the paper for example) are obtained. After the ML approach to decompose the growth rate according to its structural dependence (as encoded by $\mathbb{S}$) we can compute the softness density distribution of all crystallizing atoms, shown in Supplementary Figure 8a. This figure shows that atoms with softness values of up to $\mathbb{S} = 3$ can be found, but as we clarify below, softness values above 1.5 (i.e. beyond the maximum of the distribution) do not correspond to crystallizing atoms. These values of softness are a reflection of our limitations in devising a perfect algorithm to label the crystallization events (described in Supplementary Note 2).

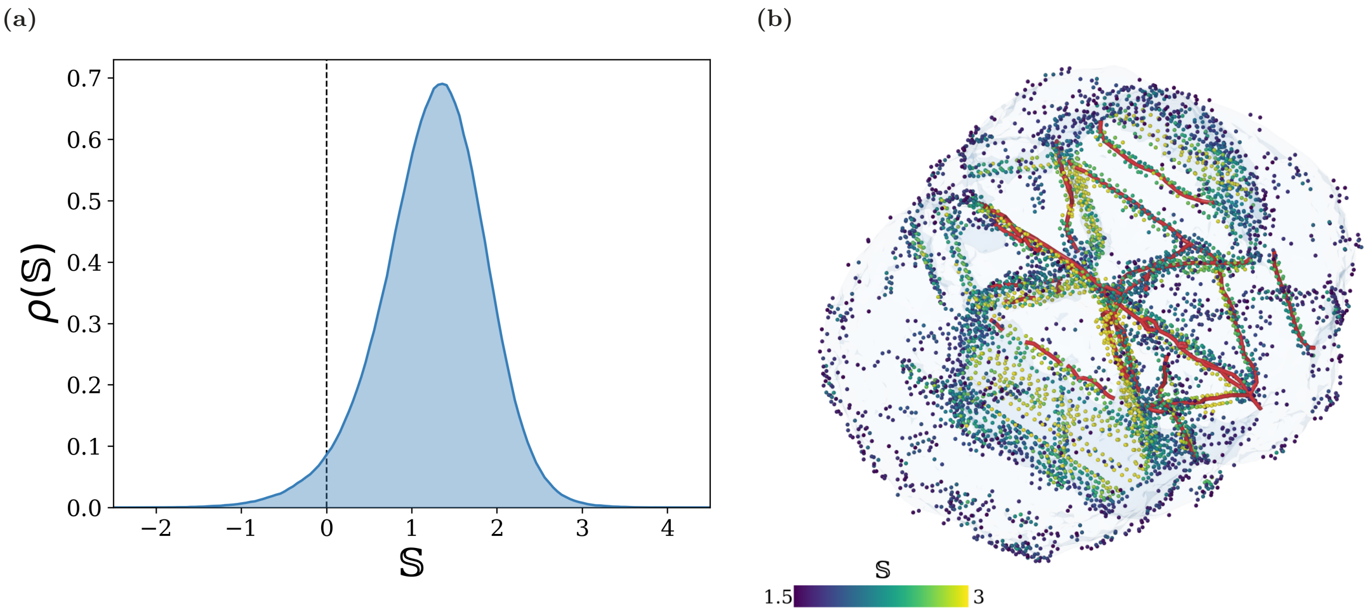

Supplementary Figure 8: **(a)** Softness density distribution. Notice the abundance of atoms with $\mathbb{S} > 1.5$ **(b)** Atoms with $\mathbb{S} > 1.5$ all appear inside the crystallite, close to defects such as dislocations (shown as thick solid lines). Hence, these are not crystallizing atoms and were excluded from the ML analysis. These observations are easier to make by watching the Supplementary Video 6. This figure was created using OVITO [15].

The origin of the $\mathbb{S} > 1.5$ finite density can be seen in Supplementary Figure 8b or, more clearly, in Supplementary Video 6. In the figure and the video atoms with softness $\mathbb{S} < 1.5$ are not shown, but we left a surface mesh to mark the boundaries between the liquid and the crystallite. It is clear in the video that while all atoms with $\mathbb{S} \approx 1.5$ lie on the crystallite interface with the liquid, the atoms with $\mathbb{S} > 1.5$ all lie inside the crystallite, and therefore they cannot be crystallizing atoms. In the figure and the video we also performed the DXA (Dislocation Extraction Algorithm [16,17]) analysis to find the dislocations inside the crystallite (shown as thick solid lines). It can be observed that the vast majority of atoms with $\mathbb{S} > 1.5$ lie around these linear defects. From the planar distribution of some $\mathbb{S} \approx 3$ atoms we also suspect that they might lie inside grain boundaries.

These observations lead us to truncate the softness distribution density (or equivalently, the growth rate decomposed into $\mathbb{S}$ shown in Fig. 4b of the paper) at $\mathbb{S} = 1.5$. We tested the effect of performing the analysis leading to Figs. 5b, 5c, and 6a of the paper with the entire softness range $\mathbb{S} \in [-1.5, 3]$ but the results for $\Delta E_{\mathrm{a}}(\mathbb{S})$, $k_0(\mathbb{S})$, and $\gamma(\mathbb{S})$ for $\mathbb{S} > 1.5$ were extremely noisy and had very large error bars, corroborating to our direct observations that $\mathbb{S} > 1.5$ atoms are not crystallizing.

Local-structure dependent model

The LSD model for the growth rate is given in Equation (2) of the paper:

$$r(T, \mathbb{S}) = k(T, \mathbb{S})\Big\{1 - \exp\big[-\beta\Delta\mu(T, \mathbb{S})\big]\Big\}. \tag{2}$$

It is shown in Fig. 5a of the paper that the kinetic factor has an Arrhenius functional form $k(T, \mathbb{S}) = k_0(\mathbb{S})\exp[-\beta\Delta E_{\mathrm{a}}(\mathbb{S})]$. It is also shown in the paper (Fig. 4a and 7a) that the LSD model is predictive in nature: after training the LSD model with data from simulations at $T \geq 1388\,\mathrm{K}$ we were able to correctly predict the temperature dependence of the growth rate for temperature as low as $1100\,\mathrm{K}$. It can also be seen in Fig. 4a that the WF model was not capable of reproducing the growth rate at temperatures for which it was not fitted to.

In this section we would like to clarify that the improved performance observed for the LSD model is not simply due to performing a parameter fitting with an additional parameter, namely softness ($\mathbb{S}$). Consider the following example to help understand why that is not the case. Suppose that instead of $\mathbb{S}$ we had introduced an arbitrary parameter $\mathbb{X}$, i.e. not derived from ML based on the local structure surrounding crystallizing atoms. The only goal of introducing $\mathbb{X}$ is to improve the fitting of the growth rate observed in the simulations. Assume for simplicity that $r(T,\mathbb{X})$ dependence on $\mathbb{X}$ follows an equation functionally identical to Supplementary Equation (2):

$$r(T,\mathbb{X}) = k_0(\mathbb{X})e^{-\beta\Delta E_{\mathrm{a}}(\mathbb{X})}\Big\{1 - \exp\big[-\beta\Delta\mu(T,\mathbb{X})\big]\Big\}.$$

According to this approach, for each value of $\mathbb{X}$ we would have one value of $\Delta E_{\mathrm{a}}(\mathbb{X})$, $k_0(\mathbb{X})$, and $\gamma(\mathbb{X})$. In the article $\mathbb{S}$ takes values from -1.5 to 1.5 in intervals of 0.05. Thus, if we fit $r(T,\mathbb{X})$ in the same interval we would have 60 independent parameters for $\Delta E_{\mathrm{a}}(\mathbb{X})$, $k_0(\mathbb{X})$, and $\gamma(\mathbb{X})$. The result of fitting the 13 points that comprise the growth rate observed in MD simulations with a function such as $r(T,\mathbb{X})$ (that depends on 180 parameters) will be overfitting. In Supplementary Figure 9 we show the result of such procedure when $r(T,\mathbb{X})$ has 12 distinct values for $\mathbb{X}$: the 13 values of growth rate observed in the MD simulation are nearly exactly reproduced by $r(T,\mathbb{X})$. Clearly, this is not the case for the LSD model. The reason for this difference is because the variables introduced by the dependence on $\mathbb{S}$ are not independent parameters for fitting: they are optimally defined using ML to discern the system kinetics based on the local structure. Thus, defining $\mathbb{S}$ in this way introduces many constraints that are not related to the reproducibility of the simulation results for the growth rate. In fact, adding $\mathbb{S}$ had not to necessarily improve the description of the growth rate at all. The fact it did affect suggests that some fundamental physics of crystal growth was captured by $\mathbb{S}$, thus improving the performance of the model in reproducing the simulation results. As we argue in the manuscript, $\mathbb{S}$ captures the dependence of the thermodynamics and kinetics of crystal growth on the microstructure (i.e. surface morphology and local liquid structure).

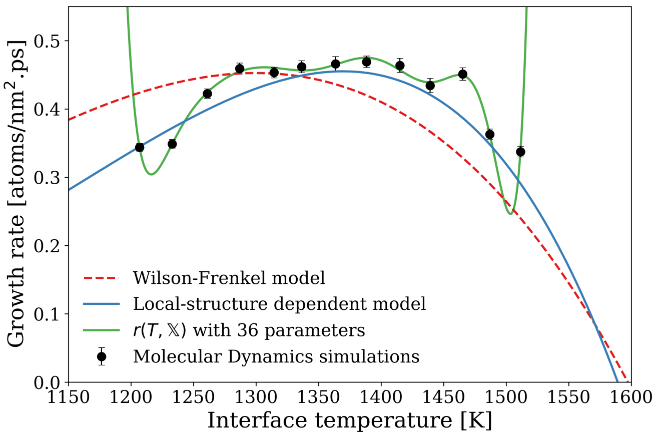

Supplementary Figure 9: The $r(T,\mathbb{X})$ model is functionally identical to the local-structure dependent model $r(T,\mathbb{S})$, but all its parameters are considered independent and were adjusted to optimally reproduce the simulation results. This demonstrates that the improved performance of the local-structure dependent model with respect to the WF model is not simply due to performing a parameter fitting of its functional form with an additional parameter. The variables introduced by the dependence on $\mathbb{S}$ are not independent parameters for fitting: they are optimally defined using ML to discern the system kinetics based on the local structure.

Supplementary Note 4: Crystallization analysis

During crystal growth the solid-liquid interface temperature is different from the temperature of the bulk liquid (or thermal reservoir) because of the release of latent heat during crystallization. Under steady-state growth conditions the solid-liquid interface temperature equilibrates at a specific value determined by the latent heat of the material and its thermal conductivity. In this section we show how the interface temperature was measured in the crystal growth simulations and how we identified when crystallization processes had achieved a steady state.

Steady-state growth

In order to determine when the growth process reaches a steady state we define the crystallite effective radius ($\mathcal{R}_{\rm eff}$) through $V = 4\pi\mathcal{R}_{\rm eff}^3/3$, where $V = N\Omega$ is the total volume of the crystallite, Ω is the volume per atom in the crystal, and N is the number of atoms in the crystallite. The calculation of N requires a criteria to select which atoms belong to the crystallite, based on the results of Supplementary Note 2 we define an atom to be in the crystallite if $\bar{\alpha}_i(t) \geq 0.25$. This criteria includes interface atoms as part of the crystallite as well.

In Supplementary Figure 10a we show the time evolution of the crystallite effective radius $\mathcal{R}_{\rm eff}$ as a function of time for simulations at different temperatures[†]. From this figure we see that the $\mathcal{R}_{\rm eff}(t)$ curve for all temperatures seem to follow the same trend: after an initial equilibration period the curve is approximately linear for a long period, then its curvature changes as it approaches a plateau when $\mathcal{R}_{\rm eff} \approx 110\,\text{Å}$ and finally it plateaus at $\mathcal{R}_{\rm eff} \approx 130\,\text{Å}$. The plateau itself only exists because of the finite size of the simulation cell (a cube with 220 Å edges), limiting and eventually stopping all crystal growth. Next we show that the linear time evolution of $\mathcal{R}_{\rm eff}(t)$ marks the period of steady state growth.

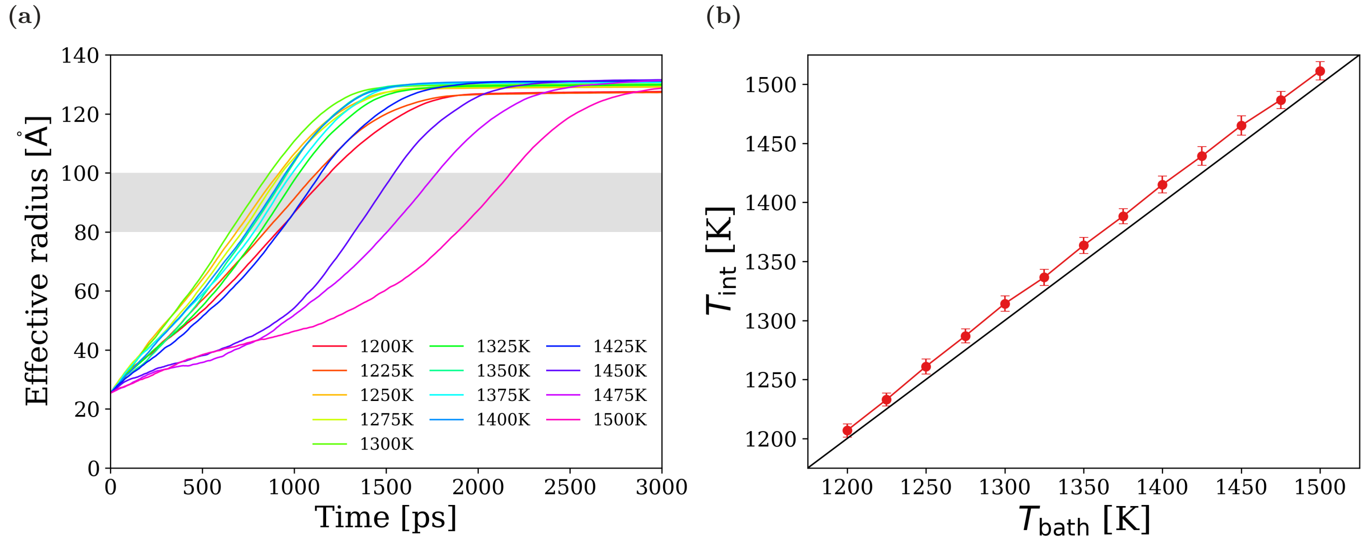

Supplementary Figure 10: **(a)** Time evolution of the effective radius of the growing crystal. The gray region marks the radius sizes considered for the calculation of the growth rate: $r_{\rm eff} \in [80\,\text{Å}, 100\,\text{Å}]$. Inside this range the crystal growth occurs in a steady-state for all temperatures considered. **(b)** Interface temperature $T_{\rm int}$ versus the thermostat bath temperature $T_{\rm bath}$ (equivalent to the liquid temperature far from the interface). The solid black line marks $T_{\rm int} = T_{\rm bath}$. The heating of the solid-liquid interface during steady-state growth is determined by a balance between the release of latent heat and the thermal conductivity of the material.

The steady-state growth condition can be defined in its most general form as when the growth rate is constant $r(t)$ = constant, i.e., when the number of attaching atoms per unit area of the crystallite interface per unit time is constant. It can be easily shown that this condition means that $\mathcal{R}_{\rm eff}(t)$ varies linearly with time:

$$\mathrm{d}V = \Omega\,\mathrm{d}N \quad \Rightarrow \quad A_{\rm eff}\,\mathrm{d}\mathcal{R}_{\rm eff} = \Omega\,\mathrm{d}N \quad \Rightarrow \quad \frac{\mathrm{d}\mathcal{R}_{\rm eff}}{\mathrm{d}t} = \Omega\frac{1}{A_{\rm eff}}\frac{\mathrm{d}N}{\mathrm{d}t} \quad \Rightarrow \quad v_{\rm eff}(t) = \Omega r(t)$$

where $r(t) = \dot{N}(t)/A_{\rm eff}(t)$ and $v_{\rm eff} = \dot{\mathcal{R}}_{\rm eff}(t)$. Thus

$$r(t) = \text{constant} \quad \Rightarrow \quad v_{\rm eff}(t) = \text{constant} \quad \Rightarrow \quad \mathcal{R}_{\rm eff}(t) = v_{\rm eff}\,t + r_0.$$

In practice we used the simulation snapshots for which $\mathcal{R}_{\rm eff} \in [80\,\text{Å}, 100\,\text{Å}]$ for all the data analysis performed in the paper and in all Supplementary Information sections. This choice of $\mathcal{R}_{\rm eff}$ was conservatively picked from the simulation

[†]Notice that all geometrical quantities related to the growth rate (such as $\mathcal{R}_{\rm eff}$) are functions of the temperature as well: $\mathcal{R}_{\rm eff}(t,T)$. Here we conceal this dependency in order to not burden the notation.

intervals where the crystal growth was in a steady state for all temperatures, as it can be seen in Supplementary Figure 10a. Then, the steady state growth rate $r(T) = \langle r(t)\rangle_{\Delta t}$, where $\langle \ldots \rangle_{\Delta t}$ denotes an average over the time interval under which the growth is in its steady state:

$$r(T) = \left\langle \frac{\dot{N}(t)}{A_{\mathrm{eff}}(t)} \right\rangle_{\Delta t}.$$

After attributing the $\mathbb{S}$ value of each crystalizing atom (i.e. each atom contributing to $\dot{N}$) according to the approach outlined in the paper we have $\dot{N}(t, \mathbb{S})$, which allow us to compute the growth rate dependence on softness:

$$r(T, \mathbb{S}) = \left\langle \frac{\dot{N}(t, \mathbb{S})}{A_{\mathrm{eff}}(t)} \right\rangle_{\Delta t}.$$

Notice in the Supplementary Video 1 that during the steady state growth (≈ 1.5 ns to 2.1 ns) the growth mechanism seems to be visibly an ordered process of islands nucleation on (111) surfaces followed by lateral growth, and also normal growth of lateral walls formed by stacking steps. This is in sharp contrast to the initial stages of growth (0 ns to ≈ 1 ns), where we still can see the (111) surfaces but we could not identify the step mechanism through visual inspection.

Interface temperature

For determining the interface temperature we first define interface atoms as all atoms with $\bar{\alpha}_i(t) \in (0.15, 0.75)$. This corresponds to atoms comprising a shell of ≈ 5 Å around the crystal. The reason we did not pick an upper bound higher than 0.75 was that when stacking fault defects were present the atoms at the defect had $\bar{\alpha}_i(t) = 0.75$. Thus, using a higher bound would incorrectly add those atoms to the analysis. Then, for each simulation temperature T the kinetic energy K_i was measured for all N_{int} interface atoms in the simulation snapshots for which the crystal growth was in a steady state (as defined in the last section). The interface temperature was computed through the energy equipartition theorem as:

$$\frac{1}{N_{\mathrm{int}}} \sum_{i=1}^{N_{\mathrm{int}}} K_i = \frac{3k_{\mathrm{B}}}{2} T_{\mathrm{int}} \qquad \Rightarrow \qquad T_{\mathrm{int}} = \frac{2}{3k_{\mathrm{B}}} \frac{1}{N_{\mathrm{int}}} \sum_{i=1}^{N_{\mathrm{int}}} K_i.$$

Figure 10b shows how T_{int} varies with the simulation temperature T.

Supplementary Note 5: Solid and liquid free energies

In Fig. 2c of the paper we show the difference in Gibbs free energy between the liquid and solid phases at zero pressure: $\Delta G(T, P=0) = G_{\mathrm{liquid}}(T, P=0) - G_{\mathrm{solid}}(T, P=0)$. Within the temperature range of the crystal growth simulations ΔG is typically smaller than 0.1 eV/atom. Thus, the solid and liquid free energies must be computed with an accuracy much better than 0.1 eV/atom in order to be used to describe the variation in growth rate as a function of temperature.

In Methods section "Liquid and solid free energies" we described how the free energy of the liquid and solid phases were computed using thermodynamic integration methods. In this section we present various methods we have employed to validate these free energy calculations.

Harmonic and quasi-harmonic approximations

First, let us define the quasi-harmonic approximation[18] (QHA) and the harmonic approximation (HA). Consider the Hamiltonian of a system of N identical particles:

$$\mathcal{H}(\mathbf{r}, \mathbf{p}) = \sum_{i=1}^{N} \frac{\mathbf{p}_i^2}{2m} + U(\mathbf{r}),$$

where $\mathbf{r} \equiv \{\mathbf{r}_1, \mathbf{r}_2, \ldots, \mathbf{r}_N\}$ are the coordinates of the N particles, $\mathbf{p} \equiv \{\mathbf{p}_1, \mathbf{p}_2, \ldots, \mathbf{p}_N\}$ are the momenta of the N particles, m is the particles' mass, and $U(\mathbf{r})$ is the many-body potential through which the particles interact with each other. In the QHA we perform a second-order Taylor expansion of $\mathcal{H}(\mathbf{r}, \mathbf{p})$ around the equilibrium lattice position of the particles: $\mathbf{r}^0(T) \equiv \{\mathbf{r}_1^0(T), \mathbf{r}_2^0(T), \ldots \mathbf{r}_N^0(T)\}$ (the temperature dependence comes from the thermal expansion of the lattice). Thus

$$\mathcal{H}(\mathbf{r}, \mathbf{p}) \approx \sum_{i=1}^{N} \frac{\mathbf{p}_i^2}{2m} + U\big(\mathbf{r}^0(T)\big) + \sum_{i=1}^{N}\sum_{j=1}^{N}\sum_{\alpha=x,y,z}\sum_{\beta=x,y,z} \frac{mD_{i,j}^{\alpha,\beta}(T)}{2}(r_{i,\alpha} - r_{i,\alpha}^0)(r_{j,\beta} - r_{j,\beta}^0), \tag{3}$$

with $r_{i,\alpha}$ the α component of ith particle's position vector $\mathbf{r}_i$ ($\alpha = x$, y, or z), and

$$D_{i,j}^{\alpha,\beta}(T) \equiv \frac{1}{m}\left(\frac{\partial^2 U}{\partial r_{i,\alpha}\partial r_{j,\beta}}\right)_{\mathbf{r}=\mathbf{r}^0(T)}$$

the potential energy Hessian matrix's components (computed here using the finite-differences method[19]). The equations of motion of the harmonic system in Supplementary Equation 3 can be uncoupled through a canonical transformation:

$$\mathcal{H}(\mathbf{r}, \mathbf{p}) \approx \sum_{n=1}^{3N} \frac{\tilde{p}_n^2}{2m} + U\big(\mathbf{r}^0(T)\big) + \sum_{n=1}^{3N}\left[\frac{1}{2}m\Omega_n^2(T)\tilde{q}_n^2\right], \tag{4}$$

where $\Omega_n(T)$ are the eigenvalues of $\mathbf{D}(T)$. The Taylor-expanded Hamiltonian, Supplementary Equation (4), is a quadratic system, hence its free energy can be computed analytically:

$$G_{\mathrm{qha}}(N, V, T) = U\big(\mathbf{r}^0(T)\big) + k_{\mathrm{B}}T \sum_{n=1}^{3(N-1)} \ln\left[\frac{\hbar\Omega_n(T)}{k_{\mathrm{B}}T}\right], \tag{5}$$

where the three null eigenvalues of $\mathbf{D}(T)$ are removed from the summation. Supplementary Equation (5) is the free energy in the QHA. Notice that in Supplementary Equation (5) the system's volume V (or, equivalently, $\mathbf{r}^0(T)$) is taken to be the equilibrium volume at temperature T and zero pressure $P = 0$.

The free energy in the harmonic approximation can be obtained from Supplementary Equation (5) by substituting $\Omega_n(T)$ by $\Omega_n(T = 0)$:

$$G_{\mathrm{ha}}(N, V, T) = U\big(\mathbf{r}^0(T)\big) + k_{\mathrm{B}}T \sum_{n=1}^{3(N-1)} \ln\left[\frac{\hbar\Omega_n(T=0)}{k_{\mathrm{B}}T}\right]. \tag{6}$$

The physical distinction between the QHA and HA is that QHA improves on the HA by incorporating anharmonic effects due to the thermal expansion of the solid.

In Supplementary Figure 11a we show the solid free energy as computed using different methods and approximations. We use as reference value the free energy computed within the harmonic approximation. It is clear in this figure that while the QHA improves on the description of the free energy with respect to the HA, there is a significant difference between the QHA and the solid free energy with no approximations (as computed by the FL and RS methods). This discrepancy is caused by anharmonic effects beyond the thermal expansion of the solid, which are expected to be significant

at temperatures close to the melting point. It is important to note here that the FL and the RS methods both account for all anharmonic effects[20].

When computing the free energy of crystalline solids the HA and the QHA are often used in the literature. These approximations allow the estimation of the solid free energy with relatively small computational costs. But, as we show in Supplementary Figure 11a, the accuracy of these methods is not enough to be used in crystal growth studies given that

$$\frac{G(T_\mathrm{m}) - G_\mathrm{qha}(T_\mathrm{m})}{k_\mathrm{B}T_\mathrm{m}} \approx 0.12,$$

where $G(T)$ is the free energy as computed using the thermodynamic integration methods. This difference results in the underestimation of the melting temperature by 92 K, as shown in Supplementary Figure 11b, which in turn results in the predicted growth rate to be up to 36% lower than its measured value. Thus, accurately accounting for all anharmonic effects is critical in crystal growth studies.

Absolute free energies

Figure 11b shows the absolute Gibbs free energy of the liquid and solid phases in the temperature range from 1100 K to 2000 K. The solid lines were computed using the Reversible Scaling[20,21] (RS) method using one of the points (as described in the Methods section "Liquid and solid free energies") as initial points for the integration. The remainder of the points were computed[20,22–25] to verify the accuracy of the RS method. The difference between the curves in Supplementary Figure 11b is $\Delta G(T, P=0) = G_\mathrm{liquid}(T, P=0) - G_\mathrm{solid}(T, P=0)$, shown in Fig. 2c of the paper.

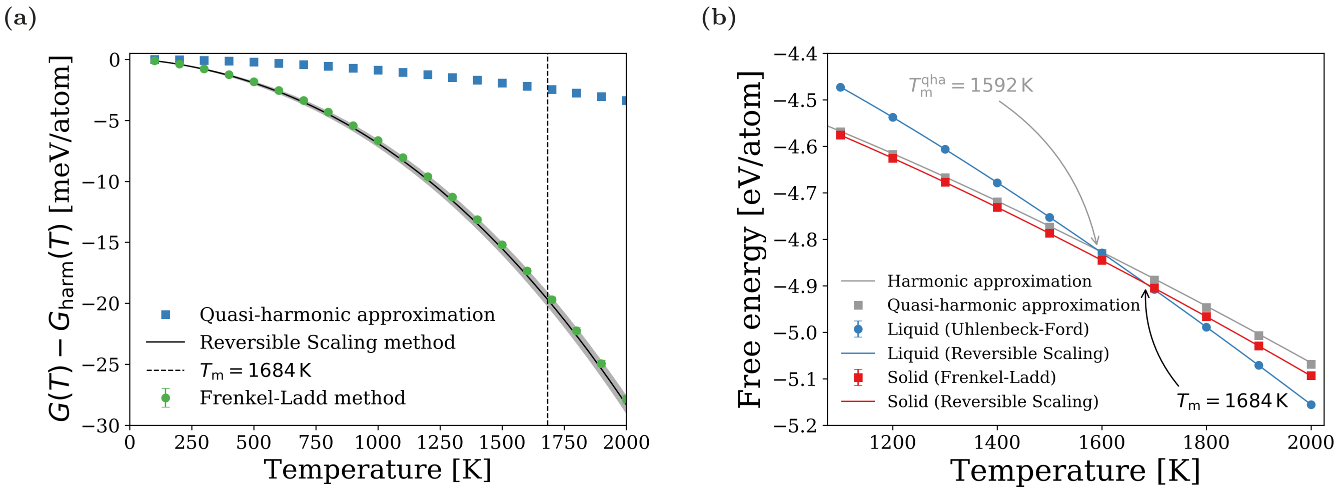

Supplementary Figure 11: **(a)** Difference in free energy with respect to the harmonic approximation. The Reversible Scaling and the Frenkel-Ladd methods capture the free energy including all anharmonic terms, while the quasi-harmonic approximation only captures anharmonic effects due to thermal expansion. **(b)** Absolute free energy curves for the liquid (in blue) and solid (in red) as computed with different methods. The harmonic and quasi-harmonic approximations (shown in gray) understimate the melting temperature by 92 K. Such understimation results in the predicted growth rate to be up to 36% lower than its measured value.

Supplementary Note 6: Liquid diffusivity

Calculating the liquid diffusivity

The liquid diffusivity was computed in a MD simulation of $8,000$ atoms in which atoms were randomly distributed in a cubic simulation cell of dimensions such that the overall density matched that of the equilibrium liquid at that temperature and zero pressure. Before the beginning of the simulation the atomic coordinates were relaxed at fixed volume by 200 timesteps using the Steepest Descent algorithm [19]. Then, initial velocities were randomly assigned from a Boltzmann distribution for the target temperature and the system was equilibrated for 10 ps using the Bussi-Donadio-Parrinello [26] (BDP) thermostat with a damping parameter of 0.1 ps. After equilibration, the thermostat was removed and the simulation was run for 100 ps, during which we measured the mean-squared displacement and the velocity autocorrelation function of all atoms:

$$[\Delta r(t)]^2 = \frac{1}{N}\sum_{i=1}^{N}[\mathbf{r}_i(t) - \mathbf{r}_i(0)]^2 \qquad \text{and} \qquad C_v(t) = \frac{1}{3N}\sum_{i=1}^{N}\mathbf{v}_i(t+t_0)\cdot\mathbf{v}_i(t_0),$$

as shown in Supplementary Figure 11a and 11b respectively. For the calculation of $C_v(t)$ the 100 ps simulation was divided into 100 intervals of 1 ps and averaged over.

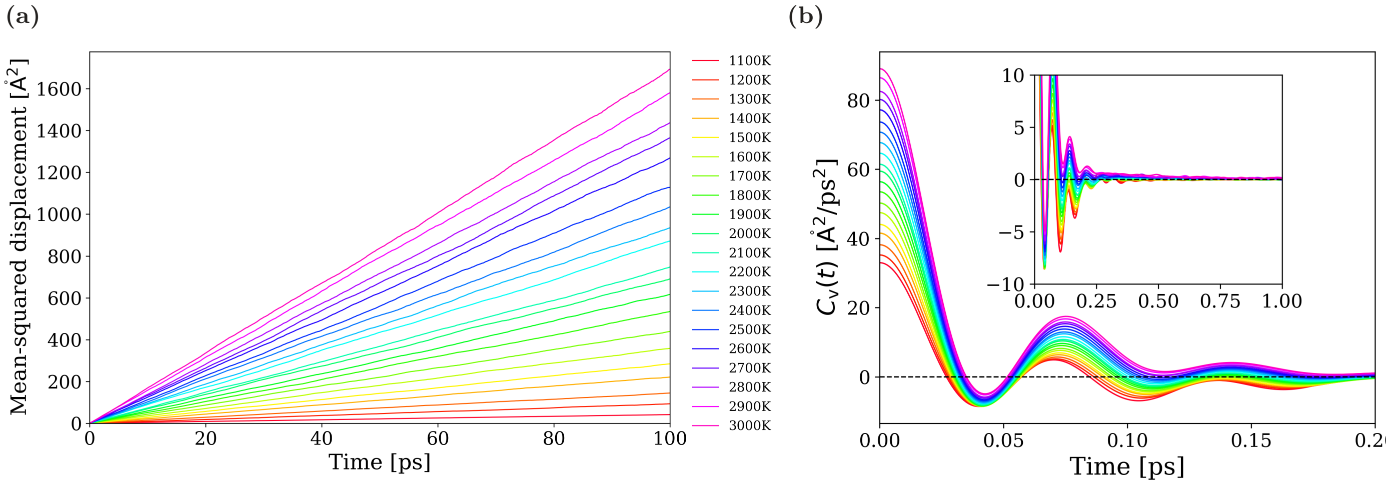

Supplementary Figure 12: Quantities used in the calculation of the liquid diffusivity. **(a)** Mean-squared displacement for different temperatures. **(b)** Velocity-velocity autocorrelation function used to compute diffusivity.

The diffusivity of the system, shown in Fig. 2b of the paper, was determined [27] as

$$D_{\text{msd}} = \frac{1}{6}\frac{\partial}{\partial t}\left\langle[\Delta r(t)]^2\right\rangle \qquad \text{and} \qquad D_{\text{vacf}} = \int_0^\infty C_v(t)\ \text{d}t,$$

where D_{msd} was computed by first making a linear approximation to $[\Delta r(t)]^2$. The diffusivity as computed by these methods agreed within the statistical uncertainty.

Thermostat parameter selection

Because the crystal growth simulations were performed with a thermostat, it is necessary to verify that the chosen thermostat parameters do no affect the kinetics of the system. The kinetics of the crystal growth process is determined by the activation energy barrier for diffusion of the liquid in the vicinity of a solid-liquid interface. Thus, we investigate here how the diffusivity of the liquid depends on the BDP thermostat damping parameter τ_{bdp}.

We have performed calculations of the diffusivity of liquid at $T = 2000\,\text{K}$ using the same system and methods described above, except that the thermostat was not removed after equilibration (i.e. before measuring the mean-squared displacement of the atoms). We have computed the diffusivity in the presence of the thermostat for different values of the thermostat damping parameter τ_{bdp}, which controls the effective timescale (or strength) of interaction between the system and the thermal bath.

Figure 13 shows that the diffusivity of the liquid is robust with respect to τ_{bdp}: $D(2000\,\text{K})$ for all considered values of τ_{bdp} agrees with the value of the diffusivity computed without a thermostat (i.e. in the microcanonical ensemble) within the error bar. Indeed, the BDP thermostat is known [26] to be excellent at reproducing the natural dynamics of the system.

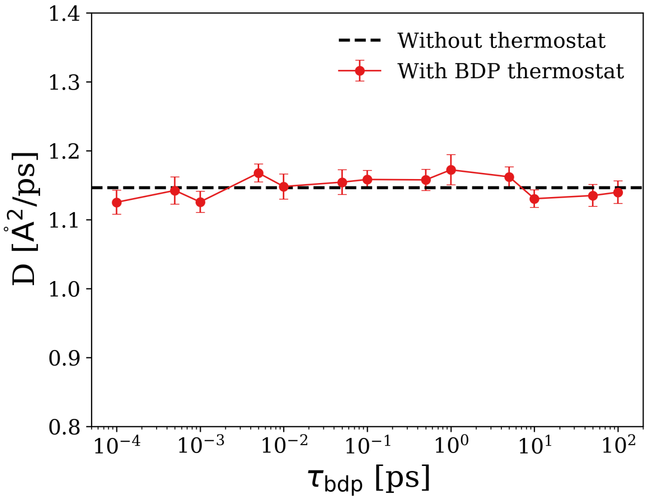

Supplementary Figure 13: Liquid diffusivity dependence on the thermostat damping parameter τ_{bdp} at $T = 2000\,\mathrm{K}$. The horizontal dashed line shows the natural liquid diffusivity in the absence of the dynamical perturbations introduced by the thermostat. The BDP thermostat is shown to be robust in reproducing the kinetics of atoms in the liquid state.

Supplementary Note 7: Results for Copper

In this section we present a collection of results for copper, all of which have a counterpart plot for silicon that has been previously discussed in details. Our goals is to make the results obtained in the paper as reproducible as possible. Note that, differently from silicon, all calculations for copper were performed without relaxing the MD snapshots because it has been shown that energy minimizations lead to significant crystalization in metallic systems[28].

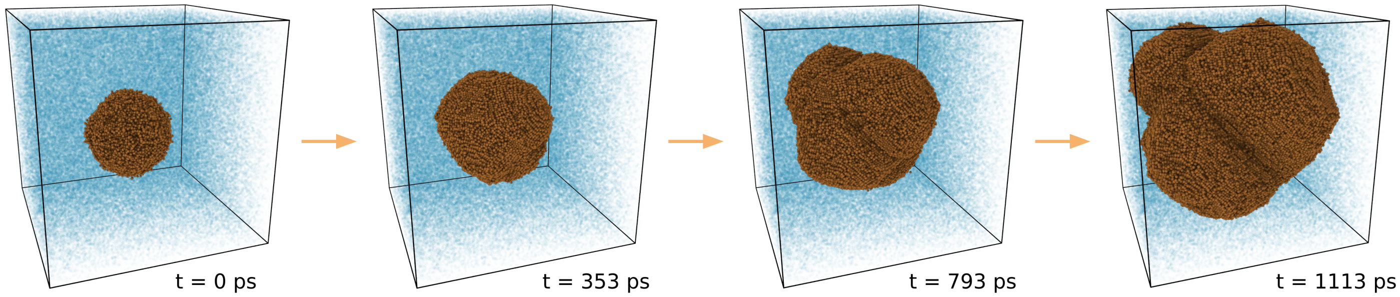

Supplementary Figure 14: Snapshots of crystal growth simulation for copper. See Supplementary Video 7 for the complete video.

Machine Learning: optimization and validation

In Supplementary Figure 15 (the corresponding information for silicon can found in Supplementary Figure 1) we show the result of the hyperparameter optimization procedure for copper. The final values for the radial structure funtion parameters for copper are quoted in the Methods section. Notice how $r_{\max}$ is almost twice as large as the value employed for silicon. We attribute this difference to the well known fact that metals present much wider solid-liquid interfaces than semiconductors.

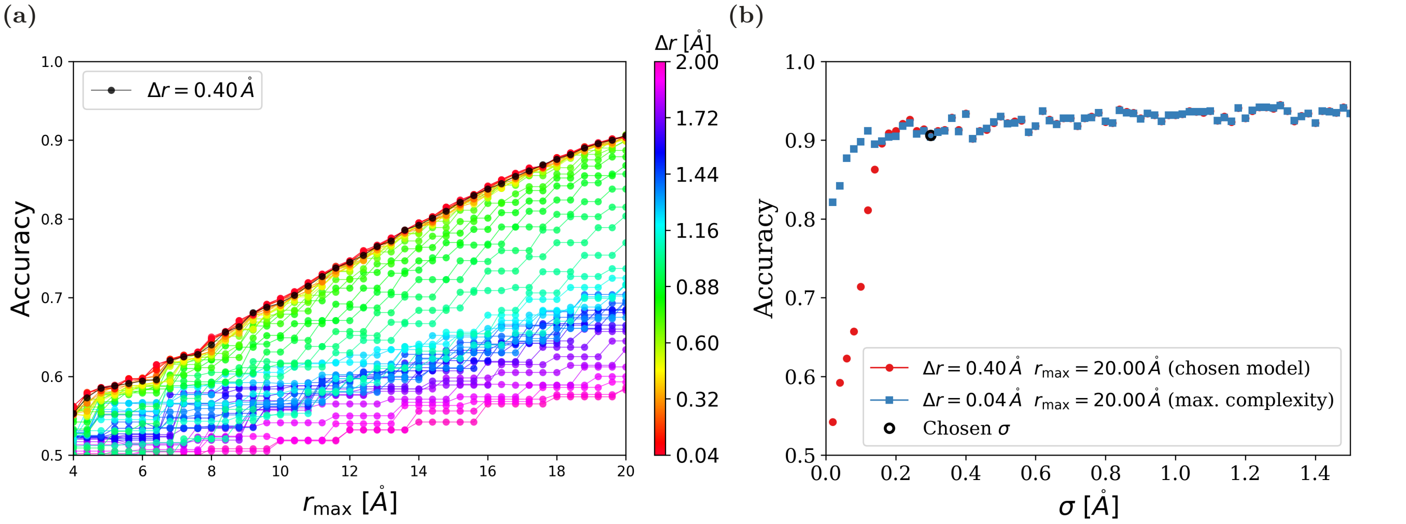

Supplementary Figure 15: Grid-search hyperparameter tuning for model feature selection for copper. **(a)** Accuracy of models with varying $r_{\max}$ and Δr for $\sigma = 0.3\,\text{Å}$. The chosen model has $\Delta r = 0.50\,\text{Å}$ (black circles) and $r_{\max} = 20\,\text{Å}$ (vertical dashed line). **(b)** Accuracy of models with different values of σ. Blue squares show the accuracy of the highest-complexity model considered (i.e. largest number of features) for comparison. Red circles show the accuracy of the chosen values of Δr and $r_{\max}$ for different values of σ.

Figure 16 (the corresponding information for silicon can be found in Supplementary Figure 2) shows the learning and validation curves for copper. Not much changes with respect to the results from silicon. Even the final classification accuracy is similar: 97%.

Growth analysis

During the steady-state growth of copper the interface temperature is up to 40 K higher than the temperature of the liquid far from the crystal, as shown in Supplementary Figure 17 (the corresponding information for silicon can be found

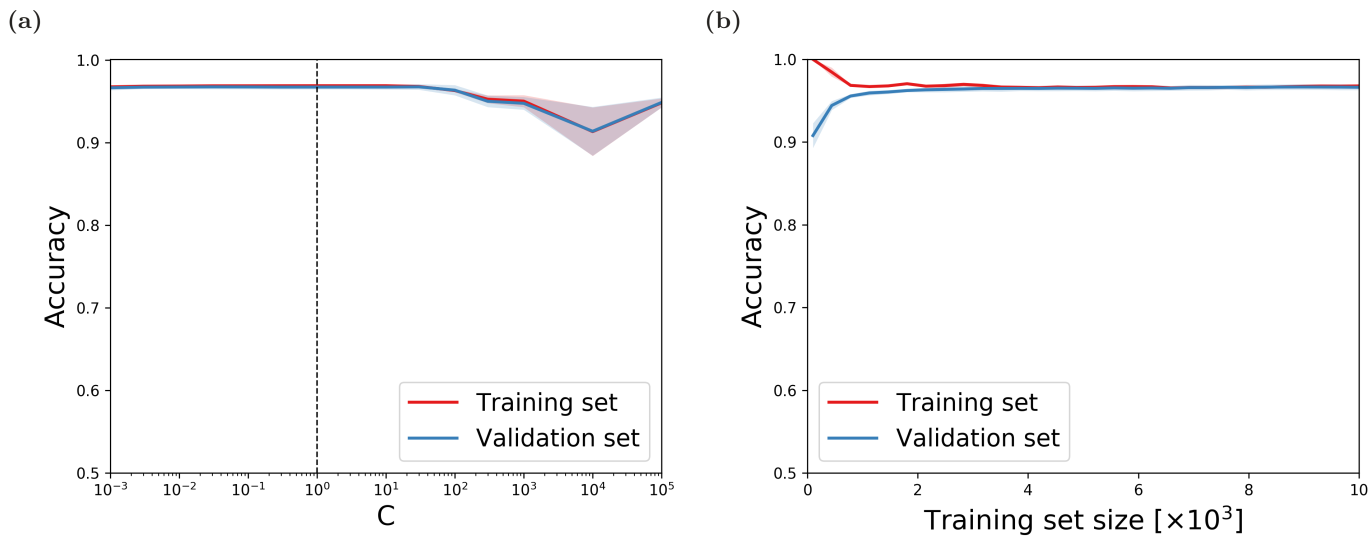

Supplementary Figure 16: Validations curves for copper. **(a)** Validation curve for SVM parameter C. Dashed line ($C = 1.0$) marks the value employed in calculations discussed in the paper. Error bars were computed using a five-fold cross-validation procedure. **(b)** Learning curve with error bars computed using a five-fold cross-validation procedure.

in Supplementary Figure 10b). This is more than twice the result from silicon. This large overheating of the interface is due to the elevated growth rate of copper, which is up to 10× faster than for silicon as can be seen in Figs. 4a and 7a from the paper.

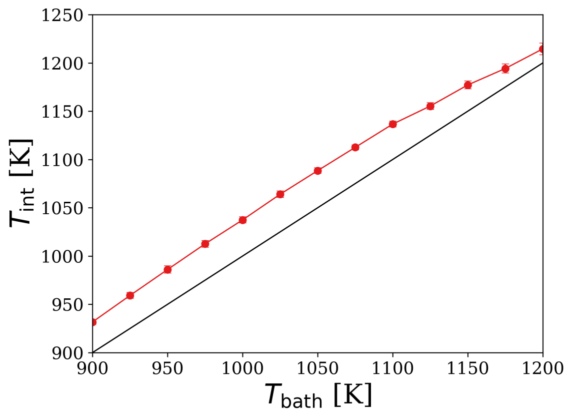

Supplementary Figure 17: Interface temperature T_{int} versus the thermostat bath temperature T_{bath} (equivalent to the liquid temperature far from the interface) for copper. The solid black line marks $T_{\mathrm{int}} = T_{\mathrm{bath}}$.

Solid and liquid free energies

The analysis of the anharmonic effects on solid copper, and the free energies of the solid and liquid phase are shown in Supplementary Figure 18 (the corresponding information for silicon can be found in Supplementary Figure 11). In particular, the quasi-harmonic approximation seems to be much more effective for copper than for silicon. Nevertheless, including all anharmonic effects is still essential to accurately model the growth rate.

Input parameters of the Wilson-Frenkel model

Input parameters for the WF model of copper are shown in Supplementary Figure 19 (the corresponding information for silicon can be found in Fig. 2b and 2c of the paper). The diffusivity of copper and silicon show similar magnitudes when plotted against T_{m}/T, and they also show similar energy barriers for diffusion ΔE_{d}. However, it is interest to notice that the crystallization driving force (ΔG) for copper decreases half as much with undercooling. This difference certainly has its origins in the difference in latent heat of fusion of these substances (13 kJ/mol for copper and 51 kJ/mol for silicon). Nevertheless, it is a curious observation that silicon presents a much larger driving force for crystallization and a much

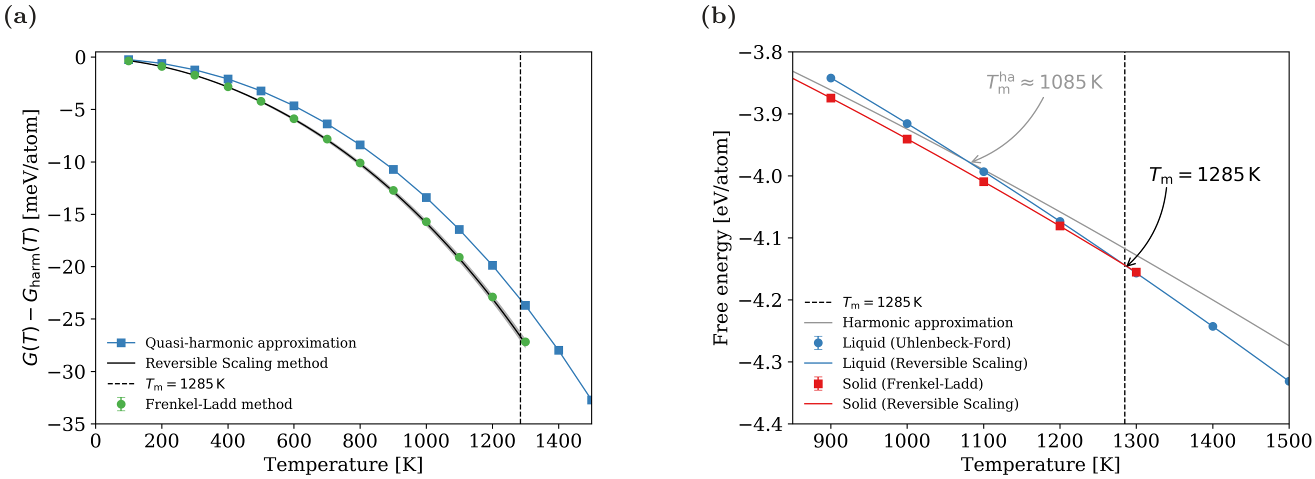

Supplementary Figure 18: Free energy of solid and liquid copper. **(a)** Difference in free energy with respect to the harmonic approximation. The Reversible Scaling and the Frenkel-Ladd methods capture the free energy including all anharmonic terms, while the quasi-harmonic approximation only captures anharmonic effects due to thermal expansion. **(b)** Absolute free energy curves for the liquid (in blue) and solid (in red) as computed with different methods. The harmonic and quasi-harmonic approximations (shown in gray) underestimate the melting temperature by ≈ 200 K.

lower growth rate. This is made even more intriguing by the fact that the activation energies for the kinetic factor of copper are much larger, as seen in Figs. 5b and 7b. This apparent conundrum is dispersed by observing that the entropic contribution for the kinetics of crystallization is many orders of magnitude larger for copper than it is for silicon. This is attributed mostly to the predominance of rough surfaces in copper that are mainly nonexistent in silicon, which strongly favors vicinal surfaces, as shown in Fig. 6a. It is slightly disappointing that this peculiar balancing of thermodynamics and kinetics has its fundamental explanation buried in the intrinsic electronic properties of these two materials.

Local-structure dependent model

Notice that the crystallization kinetic factor for copper is also observed to have Arrhenius behavior $k(T, \mathbb{S}) = k_0(\mathbb{S}) \exp[-\beta \Delta E_a(\mathbb{S})]$, shown in Supplementary Figure 19c (the corresponding information for silicon can be found in Fig. 5a of the paper).

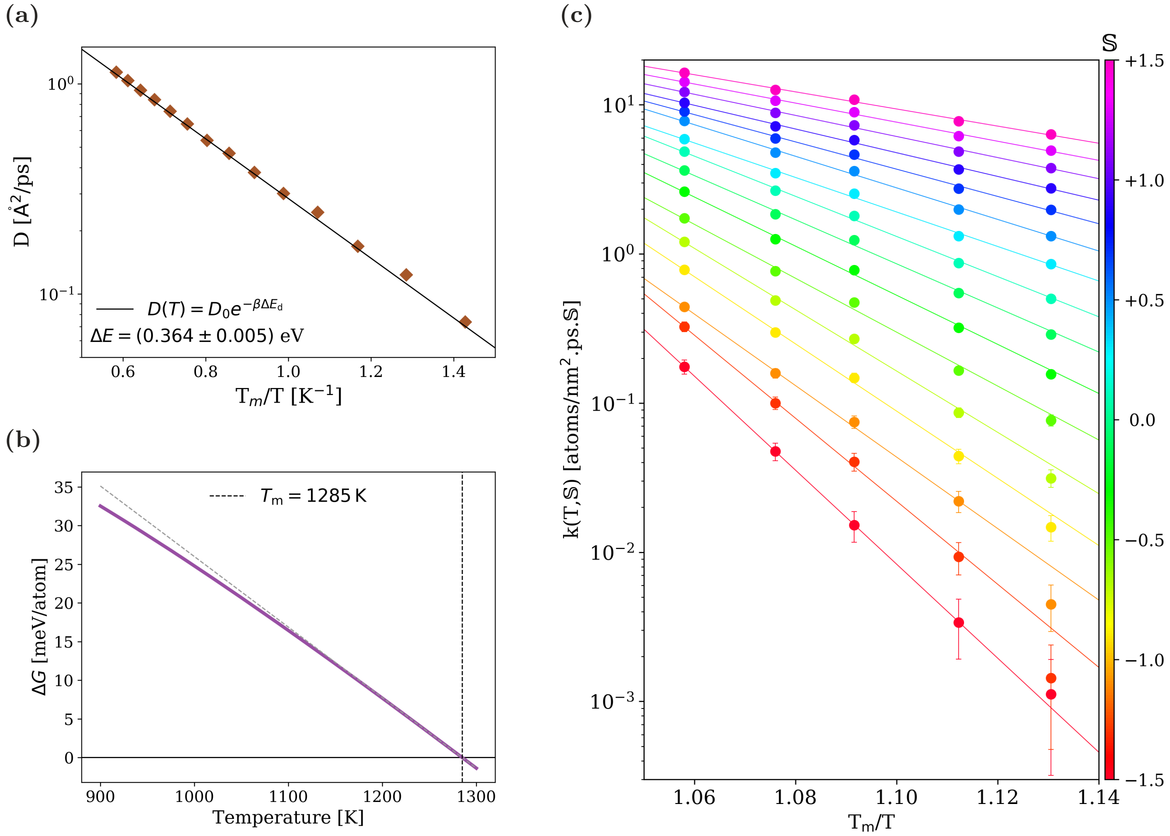

Supplementary Figure 19: Input parameters of the WF model for copper. The respective results for silicon are shown in Figs. 2b and 2c of the paper. **(a)** Arrhenius plot of the liquid diffusivity of copper as a function of temperature. **(b)** Difference in free energy between the solid and liquid phases of copper. The solid gray line is a guide to the eye to accentuate deviations from the linear behavior. **(c)** Arrhenius plot of the crystallization kinetic factor for copper. Similarly to what was observed with silicon, the kinetic factor is shown to have functional form: $k(T, \mathbb{S}) = k_0(\mathbb{S}) \exp[-\beta \Delta E_a(\mathbb{S})]$.

Supplementary References

[1] C. Cortes and V. Vapnik, "Support-vector networks," *Machine Learning*, vol. 20, pp. 273–297, 1995.

[2] F. Pedregosa, G. Varoquaux, A. Gramfort, V. Michel, B. Thirion, O. Grisel, M. Blondel, P. Prettenhofer, R. Weiss, V. Dubourg, J. Vanderplas, A. Passos, D. Cournapeau, M. Brucher, M. Perrot, and E. Duchesnay, "Scikit-learn: Machine learning in Python," *Journal of Machine Learning Research*, vol. 12, pp. 2825–2830, 2011.

[3] C.-C. Chang and C.-J. Lin, "LIBSVM: A library for support vector machines," *ACM Transactions on Intelligent Systems and Technology*, vol. 2, pp. 27:1–27:27, 2011, software available at http://www.csie.ntu.edu.tw/~cjlin/libsvm.

[4] J. Behler and M. Parrinello, "Generalized neural-network representation of high-dimensional potential-energy surfaces," *Physical Review Letters*, vol. 98, p. 146401, 2007.

[5] E. D. Cubuk, S. S. Schoenholz, J. M. Rieser, B. D. Malone, J. Rottler, D. J. Durian, E. Kaxiras, and A. J. Liu, "Identifying structural flow defects in disordered solids using machine-learning methods," *Physical Review Letters*, vol. 114, p. 108001, 2015.

[6] I. Goodfellow, Y. Bengio, and A. Courville, *Deep Learning*. MIT Press, 2016, http://www.deeplearningbook.org.

[7] J. D. Hunter, "Matplotlib: A 2d graphics environment," *Computing in Science & Engineering*, vol. 9, pp. 90–95, 2007.

[8] C.-W. Hsu, C.-C. Chang, C.-J. Lin *et al.*, "A practical guide to support vector classification," National Taiwan University, Tech. Rep., 2003. https://www.csie.ntu.edu.tw/~cjlin/papers/guide/guide.pdf

[9] J. Friedman, T. Hastie, and R. Tibshirani, *The elements of statistical learning*. Springer, 2001, vol. 1, no. 10.

[10] S. S. Schoenholz, E. D. Cubuk, D. M. Sussman, E. Kaxiras, and A. J. Liu, "A structural approach to relaxation in glassy liquids," *Nature Physics*, vol. 12, p. 469, 2016.

[11] P. Rein ten Wolde, M. J. Ruiz-Montero, and D. Frenkel, "Numerical calculation of the rate of crystal nucleation in a lennard-jones system at moderate undercooling," *The Journal of Chemical Physics*, vol. 104, pp. 9932–9947, 1996.

[12] L. v. d. Maaten and G. Hinton, "Visualizing data using t-sne," *Journal of Machine Learning Research*, vol. 9, pp. 2579–2605, 2008.

[13] L. Van Der Maaten, "Accelerating t-sne using tree-based algorithms," *The Journal of Machine Learning Research*, vol. 15, pp. 3221–3245, 2014.

[14] F. H. Stillinger and T. A. Weber, "Hidden structure in liquids," *Physical Review A*, vol. 25, p. 978, 1982.

[15] A. Stukowski, "Visualization and analysis of atomistic simulation data with ovito–the open visualization tool," *Modelling and Simulation in Materials Science and Engineering*, vol. 18, p. 015012, 2009. http://ovito.org

[16] A. Stukowski and K. Albe, "Extracting dislocations and non-dislocation crystal defects from atomistic simulation data," *Modelling and Simulation in Materials Science and Engineering*, vol. 18, p. 085001, 2010.

[17] A. Stukowski, V. V. Bulatov, and A. Arsenlis, "Automated identification and indexing of dislocations in crystal interfaces," *Modelling and Simulation in Materials Science and Engineering*, vol. 20, p. 085007, 2012.

[18] B. Fultz, *Phase Transitions in Materials*. Cambridge University Press, 2014.

[19] V. Bulatov and W. Cai, *Computer simulations of dislocations*. Oxford University Press on Demand, 2006, vol. 3.

[20] R. Freitas, M. Asta, and M. de Koning, "Nonequilibrium free-energy calculation of solids using LAMMPS," *Computational Materials Science*, vol. 112, pp. 333–341, 2016.

[21] M. de Koning, A. Antonelli, and S. Yip, "Optimized free-energy evaluation using a single reversible-scaling simulation," *Physical Review Letters*, vol. 83, p. 3973, 1999.

[22] D. Frenkel and A. J. Ladd, "New monte carlo method to compute the free energy of arbitrary solids. application to the fcc and hcp phases of hard spheres," *The Journal of Chemical Physics*, vol. 81, pp. 3188–3193, 1984.

[23] M. de Koning and A. Antonelli, "Einstein crystal as a reference system in free energy estimation using adiabatic switching," *Physical Review E*, vol. 53, p. 465, 1996.

[24] R. P. Leite, P. A. Santos-Flórez, and M. de Koning, "Uhlenbeck-ford model: Phase diagram and corresponding-states analysis," *Physical Review E*, vol. 96, p. 032115, 2017.

[25] R. P. Leite and M. de Koning, "Nonequilibrium free-energy calculations of fluids using LAMMPS," *Computational Materials Science*, vol. 159, pp. 316–326, 2019.

[26] G. Bussi, D. Donadio, and M. Parrinello, "Canonical sampling through velocity rescaling," *The Journal of Chemical Physics*, vol. 126, p. 014101, 2007.

[27] D. Frenkel and B. Smit, *Understanding molecular simulation: from algorithms to applications.* Elsevier, 2001, vol. 1.

[28] G. Sun, J. Xu, and P. Harrowell, "The mechanism of the ultrafast crystal growth of pure metals from their melts," *Nature materials*, vol. 17, p. 881, 2018.
