# Supplementary material for: Uncovering the effects of interface-induced ordering of liquid on crystal growth using machine learning
Source: Nat Commun. 2020 Jun 26;11:3260. doi: 10.1038/s41467-020-16892-4 (PMC7319977; doi:10.1038/s41467-020-16892-4)
Supplement: Supplementary file 2 — Description of Additional Supplementary Files [file 41467_2020_16892_MOESM2_ESM.pdf]

Description of Additional Supplementary Files

File name: Supplementary Video 1
Description: This video shows the entire growth trajectory of a silicon crystallite at 1500K, from the initial seed to its final size. The flat interfaces observed are all (111).

File name: Supplementary Video 2
Description: Island nucleation and growth mechanism in silicon. The video follows the nucleation and lateral growth of (111) islands. At $t \approx 1.8$ns it is possible to observe the nucleation of two islands on the same (111) flat interface, with one of them subsequently shrinking back.

File name: Supplementary Video 3
Description: Nucleation and dynamics of dislocations during the crystal growth process of silicon. Dislocations are colored by their character: blue for edge dislocations, red for pure screw dislocations, and white for mixed character. In this video all atoms were deleted, while the solid-liquid boundary was highlighted by a surface mesh around the crystallite.

File name: Supplementary Video 4
Description: In this video crystallized silicon atoms are colored in gray while crystallizing atoms are colored according to their softness value. Note how crystallizing atoms always appear near a (111) surface step or on lateral walls formed by stacking of multiple steps. This observation can be compared to the heat map of surfaces to which atoms attach to, in Fig.6a.

File name: Supplementary Video 5
Description: Same video as Supplementary Video 3 but with crystallizing atoms colored according to their softness value.

File name: Supplementary Video 6
Description: Distribution of atoms with $\mathbb{S} \geq 1.5$ in the silicon crystallite. In this video all atoms with $\mathbb{S} < 1.5$ were deleted, while the solid-liquid boundary was highlighted by a surface mesh around the crystallite. Notice how almost all atoms with $\mathbb{S} \gg 1.5$ lie inside the crystallite around dislocations. By the planar distribution of $\mathbb{S} \gg 1.5$ we also assume that some of them lie at grain boundaries and stacking faults (not shown in the video). These observations lead us to truncate the softness distribution density at $\mathbb{S} = 1.5$, since atoms with $\mathbb{S} > 1.5$ were not crystallizing atoms.

File name: Supplementary Video 7
Description: This video shows the entire growth trajectory of a copper crystallite at 1200K, from the initial seed to its final size. Notice the lack of surface steps and the predominance of rough surfaces. This is much different from silicon where the growth mechanism is step mediated, with lateral growth occurring on flat surfaces. The difference in growth mechanisms between copper and silicon

can also be observed by comparing the heat map of interfaces to which atoms attach to: fig.6b for silicon and fig.7a (inset) for Copper.

File name: Supplementary Video 8
Description: In this video the copper crystallite atoms are colored in gray while crystallizing atoms are colored according to their softness value.
